# Rating our certainty: how confidence judgments amplify belief polarization

**DOI:** 10.1007/s00426-026-02239-z

**Published:** 2026-02-07

**Authors:** Kit S. Double, Rebecca T. Pinkus, Riley Landfear, Pip Carnegie, Sophia Dowell, Micah B. Goldwater

**Affiliations:** University of Sydney, Sydney, Australia

## Abstract

Although metacognitive reflection (e.g., thinking about our confidence or certainty in a decision) is often assumed to improve decision-making, recent research suggests that eliciting confidence ratings can sometimes produce counterintuitive results, including the amplification of subjective beliefs. We report three experiments investigating how trial-by-trial confidence judgments affect the extremity of beliefs. In Experiments 1 and 2, participants provided subjective ratings of how much they liked a series of paintings and the extent to which they agreed with various social axioms, either accompanied by confidence ratings or not. Across both studies, participants who were asked to rate their confidence, gave significantly more extreme responses relative to those who did not provide confidence ratings. By contrast, in Experiment 3, when participants provided objective estimates of the age of the paintings, confidence ratings led to less extreme estimates compared to a control group. We found no evidence that the effect of eliciting confidence ratings was moderated by the average confidence associated with a judgment. We situate these findings within the broader literature on metacognition and belief polarization, discussing possible mechanisms, such as demand characteristics, selective rationalization, and cognitive biases, that may explain why confidence judgments exacerbate rather than reduce extreme subjective beliefs. We conclude with implications for interventions seeking to address polarized thinking, highlighting that confidence measurement alone may backfire unless paired with strategies to encourage genuine self-reflection and open-mindedness.

## Significance statement

Public surveys, classrooms, and social-media polls now routinely ask people not only what they think, but how sure they are. The hope is that eliciting these confidence judgments has no effect on people’s underlying beliefs. Yet that assumption has not been empirically tested. Here, we asked whether confidence prompts affect people’s beliefs and judgments. Across three experiments, participants repeatedly expressed a judgment either about personal preferences (e.g., “Do I like this painting?”) or about a verifiable fact (e.g., “When was this painting created?”). Half of the participants also rated their confidence on every trial. Confidence prompts polarized subjective opinions - people’s subjective ratings became more extreme - while simultaneously softening objective estimates. These findings show that measuring confidence in people’s beliefs may unintentionally modify those beliefs.

Belief polarization is the tendency for individuals to move toward more extreme or entrenched positions over time, which has become a pressing social concern in domains ranging from politics to health behaviors (Hutchens et al., 2019). Although there is debate as to whether people are indeed becoming more polarized (Bakker & Lelkes, 2024), the impact of polarized beliefs in everyday discourse is undeniably critical, influencing public policy, social relationships, and individual well-being (Cinelli et al., 2021; Goldenberg et al., 2023). Belief polarization is evident in domains such as climate change, vaccination attitudes, and political partisanship, where people with differing views interpret the same information in increasingly divergent ways. These examples illustrate how everyday judgments can become exaggerated through self-reinforcing certainty (Smith et al., 2024). Scholars have offered multiple explanations for the emergence of polarized thinking, pointing to echo chambers (Goldenberg et al., 2023), social media algorithms (Cinelli et al., 2021), and cognitive biases (Jern et al., 2014; Lord & Taylor, 2009).

From a psychological perspective, one of the major drivers of belief polarization is a tendency for people to engage in biased assimilation when confronted with mixed or ambiguous evidence, with individuals more readily accepting information that supports their prior belief and dismissing or critiquing information that challenges it (Lord & Taylor, 2009). This tendency to interpret information in a way that validates our pre-existing views or identity is often fueled by two related cognitive biases. Confirmation bias causes people to seek out or interpret evidence in ways that support what they already believe. At the same time, disconfirmation bias leads them to apply stricter scrutiny to evidence that contradicts their existing beliefs (Allahverdyan & Galstyan, 2014; Klayman & Ha, 1987). Notably, both biases can be reinforced by subjective *confidence* - people who feel certain about their beliefs are often more motivated to protect them, screening out counterevidence (Rollwage et al., 2018).

Confidence itself is a form of metacognitive evaluation, a second-order judgment of how certain one is in a first-order decision or belief (De Martino et al., 2013; Fleming, 2024). Importantly, confidence judgments can accompany either subjective or objective first-order decisions. Whereas objective judgments have verifiable accuracy, subjective judgments such as preferences or attitudes lack an external criterion. It has often been assumed that prompting individuals to reflect on their confidence might induce deeper processing and facilitate awareness of contradictory evidence, thus potentially *reducing* bias (Reininger et al., 2020). However, recent findings challenge this assumption: trial-by-trial confidence ratings can lengthen deliberation times without improving accuracy (Baranski & Petrusic, 2001; Double & Birney, 2024), raise decision thresholds that slow but do not correct responses (Li et al., 2024), and in some cases even impair belief updating (Double & Birney, 2018). In short, rating one’s confidence is *reactive* and can feed back into the decision process itself (Birney et al., 2017; Double & Birney, 2019). These reactive effects of confidence matter because the same metacognitive processes that shape simple perceptual decisions also influence how people interpret complex, belief-relevant information (Lebreton et al., 2015). If confidence can bias evidence processing in low-stakes tasks, it may play an even more consequential role in the formation and maintenance of polarized beliefs.

## Metacognition, polarization, and confidence

Extreme or polarized beliefs are considered detrimental because they reduce openness to evidence, intensify intergroup conflict, and undermine adaptive reasoning (Smith et al., 2024). Given the negative impacts of extreme and polarized beliefs, research has explored interventions to reduce polarized thinking (Voelkel et al., 2023). One potential approach to reducing extreme beliefs explored here involves metacognitive prompts – prompts that encourage individuals to think about their own thinking and thus recognize potential biases in their reasoning.

Participants’ metacognition is typically measured by having them make a confidence rating after each decision; here, we utilize this paradigm as a means for testing how the metacognitive reflection prompted by these ratings affects first-order ratings. Recently, several studies have shown that confidence ratings elicited during the performance of cognitive tasks influence the accuracy of the first-order decision compared to control conditions that do not make confidence ratings (Birney et al., 2017; Double & Birney, 2017, 2018, 2019, 2024; Double et al., 2025a; Li et al., 2024). Studies consistently show that when participants are asked to make confidence judgments, they take longer to make the first-order decision and respond (Baranski & Petrusic, 2001; Double & Birney, 2024; Petrusic & Baranski, 2003). However, the effect of confidence judgments on decision accuracy varies across studies: Some research has found improved performance (Double & Birney, 2017; Li et al., 2024), while other studies have found impaired performance (Double & Birney, 2024), and still others have found no effect on performance (Petrusic & Baranski, 2003).

To consider whether eliciting confidence ratings is likely to make subjective beliefs more or less extreme, we must first consider the nature of confidence in subjective beliefs. Like any other decision, our subjective beliefs and preferences are associated with a degree of confidence. For example, when we state that the Mona Lisa is a beautiful painting or that we love strawberry ice cream, each claim is associated with a sense of confidence that relates to the (un)certainty we have for the claim (Brus et al., 2021). Research in perceptual decision-making has shown that confidence can be disassociated from the decision process itself (Fleming et al., 2010), and conceptualized as reflecting a ‘second-order’ metacognitive evaluation (De Martino et al., 2013). In the case of subjective beliefs, this confidence reflects a metacognitive awareness of choice certainty (Brus et al., 2021). Recent evidence has shown that participants’ confidence increases quadratically with first-order judgments. For example, Lebreton et al. (2015) found that when rating the pleasantness of paintings, more extreme ratings (high pleasantness or high unpleasantness) were associated with greater confidence. This relationship suggests a natural synergy between subjective extremity and perceived certainty, that is people feel especially sure when their preferences are strong.

We entertain two competing hypotheses about the effect of confidence ratings on subjective beliefs. The first is that eliciting confidence ratings makes participants *less* extreme in their beliefs. By prompting metacognitive reflection while one is forming beliefs, confidence ratings may reduce the confirmation bias and prompt greater critical rethinking of one’s beliefs. Notably, people who hold more radical beliefs tend to have poorer metacognitive insight into their decisions (Rollwage et al., 2018). In addition, several studies have shown that metacognitive reflection can reduce biased thinking. For instance, Reininger et al. (2020) tested a short metacognitive training program with liberal participants in the United States to see if it would affect their attitudes toward conservatives. The results showed that participants who received this metacognitive training experienced significant reductions in their negative feelings, biased evaluations, and stereotypical thinking about conservatives. These improvements were significant when compared to participants who either received only educational information or no treatment at all. Similarly, O’Leary and Fletcher (2024) explored the effect of metacognitive reflection on belief updating. They found encouraging evidence that metacognitive reflection improved belief updating compared to a control condition who did not engage in metacognitive reflection, although the effect narrowly missed the conventional *p* <.05 threshold. Moreover, Fernbach et al. (2013) showed that when individuals attempt to explain the details of policies in greater depth, they often realize gaps in their knowledge and subsequently express more moderate views.​ However, follow-up studies noted this effect can be fragile, and replications have sometimes failed (Crawford & Ruscio, 2021), indicating it may depend on context and individual differences. The general idea, however, is that metacognitive reflection based on one’s belief (e.g., realizing one’s understanding is shallow) can reduce unwarranted certainty.

It is important to distinguish between metacognitive prompts that require articulation of reasoning or knowledge (e.g., explaining policy positions or stereotypes, as in Fernbach et al., 2013; Reininger et al., 2020), and simpler metacognitive judgments like confidence ratings which merely ask for a self-assessment of certainty. The former may facilitate belief calibration by surfacing knowledge gaps, while the latter may merely reinforce existing intuitions without critical scrutiny. This distinction could help explain why some metacognitive interventions reduce polarization while others exacerbate it. There is also indirect evidence that confidence ratings might prompt more conservative thinking from studies looking at reactivity to confidence ratings in perceptual decision-making. Li et al. (2024) found that participants who made confidence ratings were more conservative in their responding on a cognitive task than their peers in the control condition. Specifically, they used a drift-diffusion model to show that confidence ratings increased participants’ decision thresholds such that they required greater evidence before making a decision in the perceptual task.

However, there is also evidence that might suggest that eliciting confidence ratings may actually increase extreme beliefs. Several studies suggest that eliciting metacognitive ratings decreases participants’ willingness to engage in deep reflective thinking. Double et al. (2025a) demonstrated that when asked to make metacognitive ratings, participants tend to choose more expedient cognitive strategies and avoid those requiring deeper processing. This finding is consistent with Mitchum et al. (2016), who observed that participants making metacognitive ratings allocated more time to learning easier items while spending less time on difficult ones, suggesting an aversion to engaging with more challenging cognitive stimuli.

Furthermore, Double and Birney (2018) found that participants who provided confidence ratings during cognitive tasks showed reduced belief updating about their own abilities. Their retrospective performance appraisals aligned more closely with their pre-task predictions than control participants and less closely tracked their actual performance. This indicates that participants making metacognitive ratings were less likely to revise their beliefs based on new evidence. Collectively, these findings suggest that rather than promoting critical reflection, eliciting metacognitive ratings may have a counterproductive effect, leading to the reinforcement and intensification of participants’ existing beliefs. Together, these considerations suggest a potential asymmetry in confidence reactivity: for subjective tasks where beliefs are affectively laden but not knowledge-calibrated, confidence ratings may reinforce extremity. For objective tasks, they may encourage caution by increasing decision thresholds or highlighting uncertainty. The present experiments were designed to test this asymmetry.

### Present experiments

In the current set of experiments, we examine the effect of eliciting confidence ratings on subjective beliefs. In each experiment, participants make a response using a Likert-like scale, after which they either make a confidence rating or immediately continued to the next item. In Experiment 1, participants make ratings of the likeability of paintings. In Experiment 2, participants rate their agreement with social axioms. Experiment 3 employed an objective estimation task (dating each painting). In each experiment, we measure the extremeness of the response by modeling the quadratic (squared) response value.

We selected liking of paintings and social axioms because they represent subjective judgments of increasing cognitive entrenchment. Painting preferences are typically flexible, aesthetic judgments, while social axioms reflect broader worldview assumptions. Neither is likely to invoke strong identity-protective reasoning, but they differ in presumed stability. We expected that if confidence ratings affect polarization through response consolidation or self-justification, this should occur across both types of subjective judgment. In contrast, the dating task in Experiment 3 involves objective estimation where participants lack strong priors or identity-driven stakes. Here, metacognitive prompts might promote more conservative responding, as found in prior perceptual decision studies (Li et al., 2024). Finally, we will perform a cross-experiment analysis where we examine whether the effect of eliciting confidence ratings is better explained by the nature of the task (subjective versus objective) or the average confidence participants have in their judgment. Additionally, in each experiment we examine whether the effect of eliciting confidence ratings is moderated by the average confidence associated with the judgment.

## Experiment 1

### Method

#### Participants

The studies were approved by the Human Research Ethics Committee of an Australian University. Participants were recruited from Prolific Academic (www.prolific.com). Participation was limited to participants residing in the United States or the United Kingdom and participants were paid £0.70 for completing the study. The sample size was determined by a power analysis using G*Power (Faul et al., 2007) performed using an independent samples *t*-test, *d* = 0.40, based on the typical effect sizes observed in previous work on reactivity to confidence ratings (Birney et al., 2017; Double & Birney, 2017; Double et al., 2025a). The analysis indicated that a minimum sample of 200 participants was required to achieve 80% power.

A total of 200 participants were included in the study (50.5% female, *M*_age_ = 37.95, *SD* = 12.43). Participants were randomly allocated to a condition that made confidence ratings (CR condition; *n* = 101) or a control condition that did not make confidence ratings (No-CR condition; *n* = 99).

#### Materials and procedure

The experiment was programmed in jsPsych. The task was based on the task used by Lebreton et al. (2015) and required participants to rate the likeability of a series of paintings. Paintings were drawn from the website of The Metropolitan Museum of Art. 120 paintings were selected from their repository. All paintings were painted between the years 1500 and 2000. Available details of the paintings (e.g., artist, completion date) are available on the Open Science Framework repository (https://osf.io/ju73w/).

**Fig. 1:**
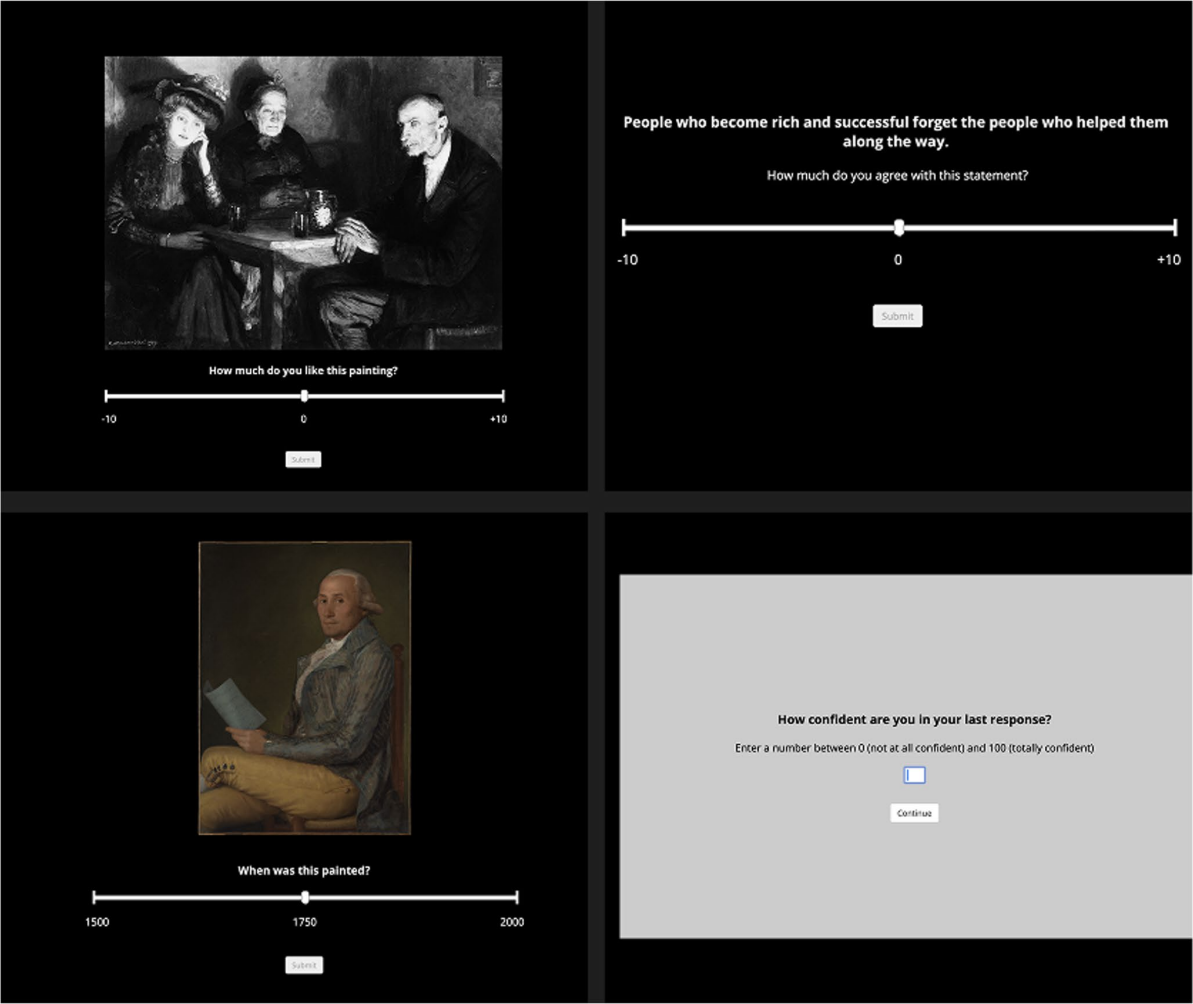
Experimental Stimuli. (Top Left) Likeability ratings used in Experiment (1) (Top Right) Agreement ratings used in Experiment (2) (Bottom Left) Dating estimates used in Experiment (3) (Bottom Right) Confidence ratings used in all experiments

Participants rated 30 of the 120 paintings which were chosen at random for each participant. As shown in Fig. 1, on each trial a painting was displayed, and participants were asked ‘How much do you like this painting?’. Participants had unlimited time to make their responses. Reaction time was defined as the duration of the first-order response (e.g., liking or agreement rating) and did not include the subsequent confidence entry, ensuring comparability between confidence-rating and control groups. Participants answered the question on a 21-point scale ranging from − 10 to + 10 with a 0 midpoint. A 21-point scale was employed to match prior paradigms looking at how extreme responses relate to confidence (e.g., Lebreton et al., 2015) and to capture fine-grained variation in response extremity. This wide scale facilitates modeling of quadratic effects while maintaining interpretability for participants.

After each response, participants in the No-CR condition simply progressed to the next item, while participants in the CR condition made a confidence rating. The confidence rating asked ‘How confident are you in your last response?’. Participants were told to enter a number between 0 (*not at all confident*) and 100 (*totally confident*). We used a different response modality (numerical entry) than the primary response to minimize dependency between the first-order response and the confidence rating. Participants had unlimited time to make their confidence rating.

### Results and discussion

Prior to analysis, we performed several checks to ensure data quality. We removed responses with overly long response times (which we defined as being more than 3 standard deviations above the mean) and removed confidence ratings outside of the required [0, 100] range. To parametrize response polarization, we squared participants’ responses on the − 10 to + 10 likeability scale.

**Fig. 2:**
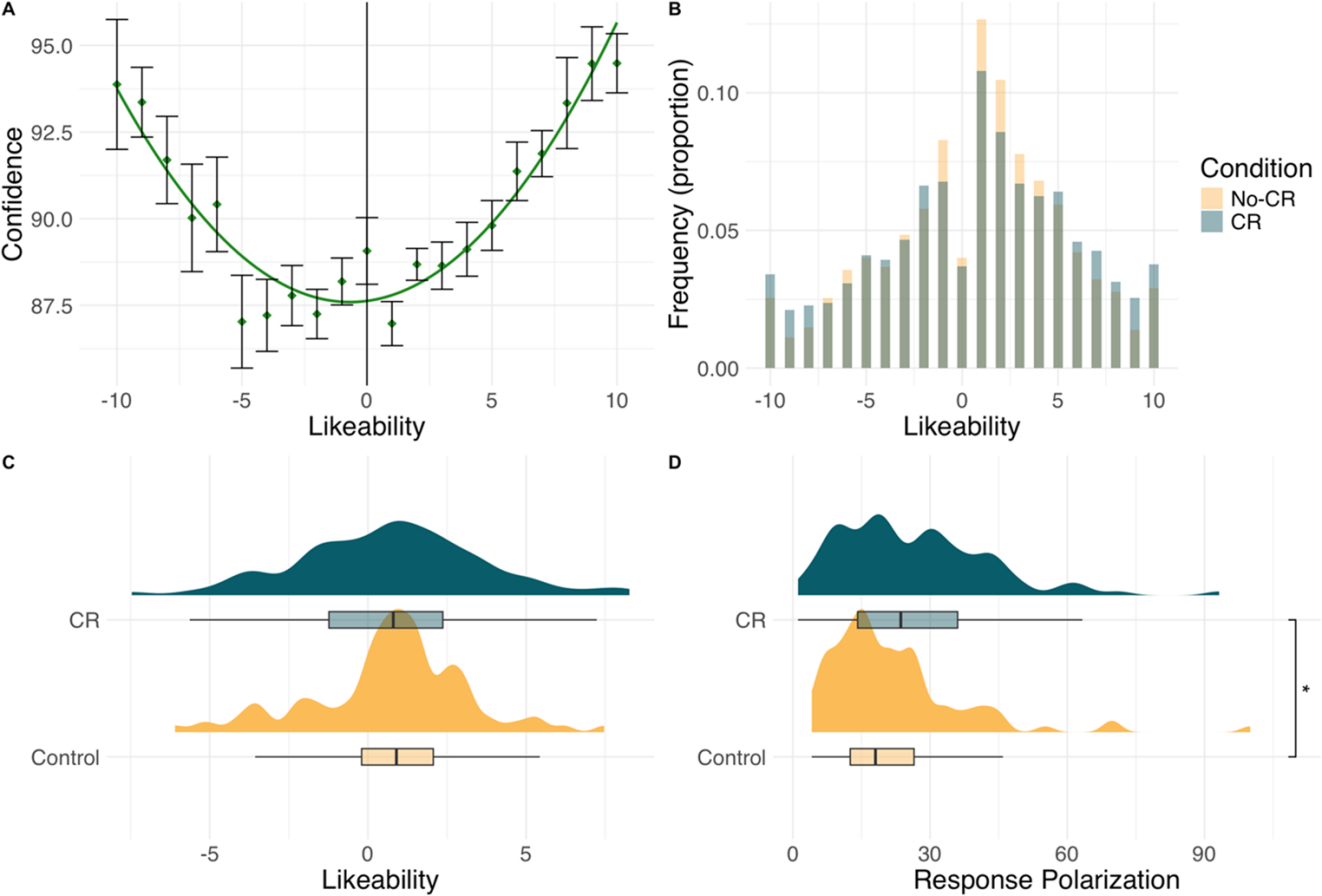
Response distributions in Experiment 1. (**a**) confidence ratings vary as a U-shaped function of likeability. Error bars = ± 1 SE (**b**) frequency of likeability responses as a function of experimental condition. (**c**) distributions of likeability as a function of experimental condition. (**d**) Response polarization (squared likeability responses) as a function of experimental condition

We first analyzed the relationship between confidence ratings and likeability ratings. This response was necessarily limited to participants in the CR condition. As previous studies have shown a U-shaped (quadratic) relationship between confidence and likeability responses, we modeled the relationship using a linear effects model with random intercepts for each participant. The second-order orthogonal polynomials of likeability (linear and quadratic terms) were used to predict participants’ confidence. The model indicated there was a significant positive linear relationship between likeability and confidence (*b* = 53.44, SE = 12.86, *p* <.001), suggesting that participants were more confident at higher positive or negative likeability rating. Furthermore, the quadratic term was also a significant positive predictor of confidence (*b* = 164.53, SE = 12.62, *p* <.001). As shown in Fig. 2a, we replicated the previously observed U-shape relationship between likeability ratings and confidence (Lebreton et al., 2015).

Likeability ratings of participants in the CR condition (*M* = 0.71, *SD* = 5.09) were not significantly different from those of participants in the No-CR condition (*M* = 0.69, *SD* = 4.64), *t*(198) = 0.06, *p* =.953, *d* = −0.008. However, response polarization was significantly greater in the CR condition (*M* = 26.46, SD = 30.09) than in the No-CR condition (*M* = 21.98, *SD* = 27.23), *t*(198) = 2.01, *p* =.046, *d* = 0.28. Analyses are illustrated in Fig. 2.

Additionally, we analyzed whether there were differences in response times as a function of condition. The average response times of participants in the CR condition (*M* = 5929ms, *SD* = 3762) were not significantly different from those of the control condition (*M* = 5421ms, *SD* = 3455), *t*(198) = 1.32, *p* =.187, *d* = −0.19.

Finally, we consider whether the confidence associated with a particular judgment moderates the effect of eliciting confidence ratings. While exploring this possibility would be straightforward if we had confidence ratings from all participants, it is made more complex because we do not know the confidence of participants in the control condition. To circumvent this issue, we therefore need to work out the average confidence associated with a particular stimulus (and assume this is representative of both the confidence rating and control groups). Note, we also carried out this analysis using the median of participants’ confidence ratings with the pattern of the results unchanged. Mean confidence ratings in the confidence-rating condition were 89.51 (SD = 11.88), indicating moderate variability across participants. These values provide a context for the stimulus-level confidence proxy used in moderation analyses. After calculating the average confidence for each stimulus, we entered it as a moderator of the condition effect (CR versus No-CR) in a multilevel model predicting response polarization. The model indicated that confidence did not significantly moderate the effect of eliciting confidence ratings (*b* = 0.28, SE = 0.23, *p* =.226).

Although mean agreement ratings did not differ significantly between conditions, polarization scores were higher in the confidence-rating condition because the quadratic transformation captures the extremity of ratings irrespective of direction. This indicates greater dispersion toward scale endpoints rather than a shift in mean position. Taken together, these results suggest that confidence increased response polarization for likeability ratings.

## Experiment 2

The findings from Experiment 1 suggest that eliciting confidence ratings produced higher belief polarization when participants rate the extent to which they like paintings. While this provides initial evidence that subjective beliefs are reactive to confidence ratings, likeability ratings are a relatively superficial and flexible belief and thus are potentially more malleable (and susceptible to reactivity) than more deeply held subjective beliefs that are more entrenched. We therefore use Experiment 2 to examine whether participants’ agreement with various social beliefs, which are likely to be pre-existing and more stable, are also reactive to confidence ratings.

### Method

#### Participants

Participants were recruited in the same fashion as Experiment 1. 200 participants (48.5% female, *M*_age_ = 36.78, *SD* = 12.17) were included in the study based on the power assumptions used in Experiment 1. As in Experiment 1, participants were randomly allocated to a CR condition (*n* = 105) or a No-CR condition (*n* = 95).

#### Materials/Procedure

Rather than rating the likeability of paintings, participants were required to rate their agreement with various social axioms.

##### Social Axioms Survey (Tong et al., 2023)

Participants completed the 20-item short-form of the Social Axioms Survey which asks participants to rate their agreement with generalized beliefs and broad assumptions about the world, for example, ‘*A person’s behavior is determined by many factors*’ or ‘*Individual characteristics*,* such as appearance and birthday*,* can reveal one’s fate’.* We utilized the same 21-point response scale from Experiment 1. Participants were asked “How much do you agree with this statement?”. After rating each statement, participants allocated to the CR condition were required to make a confidence rating in the same manner as Experiment 1.

### Results and discussion

**Fig. 3:**
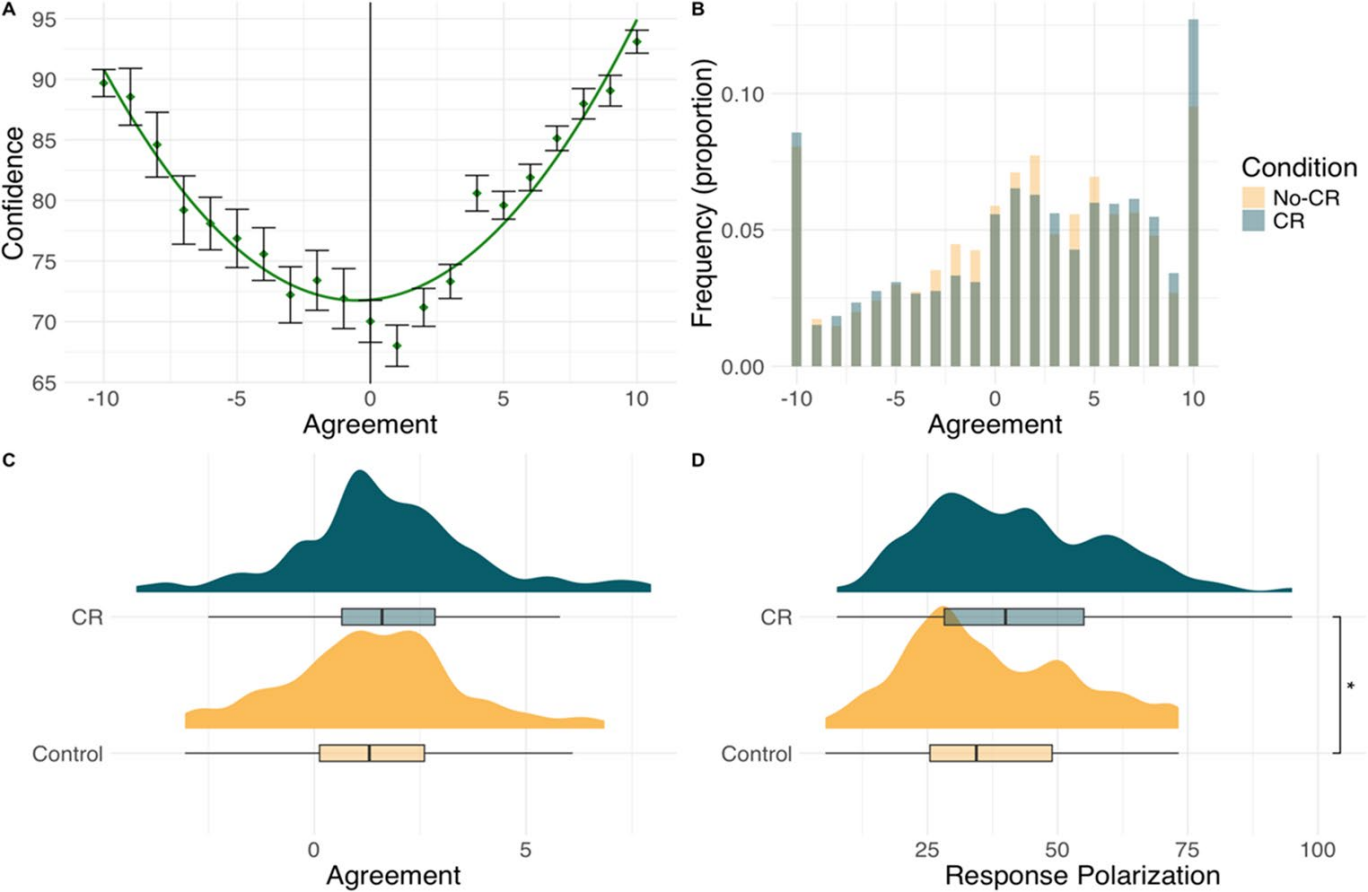
Response distributions in Experiment 2. (**a**) confidence ratings vary as a U-shaped function of agreement. Error bars = ± 1 SE (**b**) frequency of agreement as a function of experimental condition. (**c**) distributions of agreement as a function of experimental condition. (**d) **Response polarization (squared agreement responses) as a function of experimental condition

We first performed the same data screening detailed in Experiment 1. We then modeled the relationship between confidence and agreement in the same fashion as the confidence-likeability relationship in Experiment 1. The model indicated there was a significant positive linear relationship between agreement and confidence (*b* = 115.47, SE = 16.57, *p* <.001), suggesting that participants were more confident as agreement ratings increased. Furthermore, the quadratic term was also a significant positive predictor of confidence (*b* = 449.97, SE = 17.42, *p* <.001). As shown in Fig. 3a, the relationship replicated the U-shape observed in Experiment 1.

Agreement of participants in the CR condition (*M* = 1.72, *SD* = 2.25) was not significantly different from that of participants in the No-CR condition (*M* = 1.39, *SD* = 2.04), *t*(198) = 1.10, *p* =.271, *d* = −0.16. However, response polarization was significantly greater in the CR condition (*M* = 41.90, *SD* = 17.34) compared to the No-CR condition (*M* = 36.96, *SD* = 16.26), *t*(198) = 2.07, *p* =.039, *d* = −0.29. Analyses are illustrated in Fig. 3. These findings replicated Experiment 1 in suggesting that eliciting confidence ratings increased response polarization by prompting more extreme responding.

Moreover, we analyzed whether there were differences in response times as a function of condition. Unlike Experiment 1, the average response times of participants in the CR condition (*M* = 6284ms, *SD* = 2157) were significantly slower than those in the control condition (*M* = 5645ms, *SD* = 1801), *t*(198) = 2.26, *p* =.025, *d* = −0.32.

Finally, we again consider whether the confidence associated with a particular judgment moderates the effect of eliciting confidence ratings using the same model as Experiment 1. Mean confidence ratings in the confidence-rating condition were 80.60 (SD = 15.11), indicating moderate variability across participants The model indicated that confidence did not significantly moderate the effect of eliciting confidence ratings (*b* = 0.13, SE = 0.22, *p* =.541).

These results suggest that, like likeability ratings, confidence increased response polarization for agreement ratings on the social axiom scale. Interestingly, participants in the confidence-rating condition showed slower response times, suggesting that the prompt may have introduced additional metacognitive load or reflective deliberation rather than immediate self-justification. This pattern indicates that confidence prompts can engage reflective processing even within subjective domains, supporting the view that confidence reactivity may depend on whether prompts encourage analytical versus confirmatory reflection.

## Experiment 3

The previous experiments suggested that eliciting confidence ratings from participants when they made a subjective rating about a stimulus (likeability, agreement) enhanced response polarization. Although these findings may indicate a genuine increase in extreme sentiment, it is unclear if the confidence ratings are simply biasing the way participants use the scales. While we have no reason to suppose that confidence ratings nudge people towards the more extreme values of a scale, we designed Experiment 3 to test whether we would observe the same response polarization when participants were making a more objective judgment instead of a subjective judgment. Presumably, if rather than producing more extremely subjective beliefs, confidence ratings are actually biasing participants’ use of the response scale more generally, then we should not observe increases in polarization when confidence ratings are elicited for a task involving more objective ratings. To this end, Experiment 3 examines whether we observe greater response polarization when participants are asked to estimate the age of the same paintings used in Experiment 1.

### Method

#### Participants

Participants were recruited in the same manner as in the previous experiments. 200 (50% female, *M*_age_ = 38.69, *SD* = 13.88) were included in the study. 101 participants were randomly allocated to the CR condition and 99 participants were randomly allocated to the No-CR condition.

#### Materials/Procedure

The same procedure outlined in Experiment 1 was used with the exception that participants were asked to estimate the year the painting was completed (rather than likeability). The prompt ‘*When was this painted?’* was shown under each painting. Participants had to select the year on a 21-point scale ranging from 1500 to 2000 with a midpoint (1750) displayed, see Fig. 1. As in the previous studies, those in the CR condition immediately made the same confidence rating following their rating.

#### Results and discussion

**Fig. 4:**
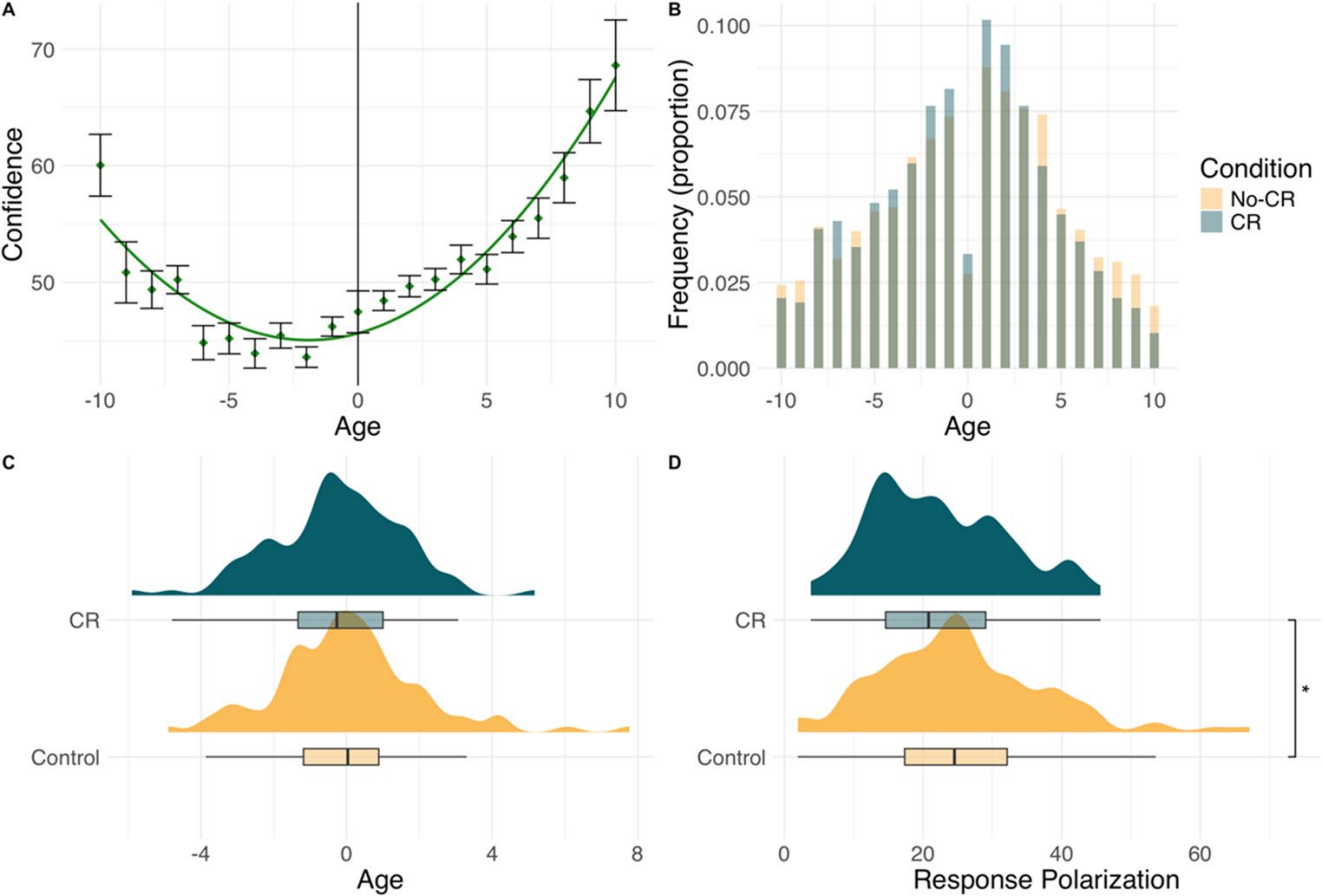
Response distributions in Experiment 3. (**a**) confidence ratings vary as a U-shaped function of age estimates. Error bars = ± 1 SE (**b**) frequency of age estimates as a function of experimental condition. (**c**) distributions of date estimates as a function of experimental condition. (**d**) Response polarization (squared date estimates) as a function of experimental condition

After performing the same data checks used in the previous experiments, we modeled the linear and quadratic relationship between age ratings and confidence using a linear effects model. The model indicated there was a significant positive linear relationship between date estimates and confidence (*b* = 152.15, SE = 16.54, *p* <.001), suggesting that participants were more confident as age estimates increased. In addition, the quadratic term was again a significant positive predictor of confidence (*b* = 229.49, SE = 16.42, *p* <.001). The relationship again replicated the U-shape observed in the previous experiments, see Fig. 4.

The date estimates of participants in the CR condition (*M* = −0.25, *SD* = 1.79) were not significantly different from those of participants in the No-CR condition (*M* = 0.07, *SD* = 2.02), *t*(198) = 1.20, *p* =.232, *d* = 0.17. To calculate response polarization in the same fashion as the previous experiments, we first converted the age estimates so that responses reflected the underlying 21 scale points that were available to participants. Thus, as with the previous studies, the midpoint (1750) was set to zero on a scale with a range of −10 to + 10. We then squared this response to calculate response polarization. There was a significant difference in response polarization between the No-CR condition (*M* = 25.44, *SD* = 12.13) and the CR condition (*M* = 22.24, *SD* = 9.63), *t*(198) = 2.07, *p* =.040, *d* = 0.29. This suggests that participants in the No-CR condition made more extreme estimates of the painting’s age (older or younger) than those in the CR condition.

As the age of the paintings are objective estimates, we were able to compare the accuracy of the date estimates between the two conditions. To compute accuracy, we took the absolute value of the difference between a participant’s estimated date of the painting and the actual completion date of the painting. The difference between the No-CR (*M* = 102.49, *SD* = 29.56) and CR conditions (*M* = 96.56, *SD* = 25.14) was not significant, *t*(198) = 1.53, *p* =.128, Cohen’s *d* = 0.22.

Next, we analyzed whether there were differences in response times as a function of condition. The average response times of participants in the CR condition (*M* = 7590ms, *SD* = 3535) were not significantly different from those in the control condition (*M* = 7127ms, *SD* = 3880), *t*(198) = −0.88, *p* =.379, *d* = −0.12.

Finally, using the same model as the previous experiments, we tested whether the average confidence associated with a stimulus moderated the effect of eliciting confidence ratings. Mean confidence ratings in the confidence-rating condition were 49.25 (SD = 18.46), again indicating moderate variability across participants. The model again indicated that confidence did not significantly moderate the effect of eliciting confidence ratings (*b* = 0.11, SE = 0.11, *p* =.294).

Overall, our results suggest that, unlike the previous studies, when participants made an objective rating (dating the paintings), confidence ratings produced significantly *less* belief polarization.

### General discussion

Across three experiments, eliciting confidence judgments had divergent effects on subjective versus objective ratings. In Experiments 1 and 2, confidence prompts led to greater polarization in *likeability* and *agreement* ratings, whereas in Experiment 3, they slightly *reduced* the extremity of *age* estimates. These findings illustrate that the reactivity of confidence judgments (Birney et al., 2017; Double & Birney, 2019) can manifest differently depending on whether beliefs are primarily *preference-based* or *fact-based*.

#### Why would confidence ratings amplify extremity in subjective tasks?

One potential explanation for why confidence ratings produce more extreme subjective ratings, is that asking for confidence “locks in” participants’ immediate subjective response. When people make a confidence rating, they might search for internal consistency and self-justify their initial reaction, thereby pushing them toward an extreme end of the scale on subsequent responses (Double & Birney, 2018). Additionally, confident stances are socially valued in many cultures, so participants could interpret the confidence prompt as a cue to “stand by” their initial impression. This phenomenon aligns with post-decision rationalization (Lind et al., 2017) and post-decisional dissonance effects (cf. Festinger, 1957) and could reflect a broader mechanism in which a confidence rating amplifies existing biases, including confirmation bias and disconfirmation bias (Lord & Taylor, 2009).

Another factor is that subjective beliefs and preferences already exhibit a natural correlation between extremity and confidence (Brus et al., 2021; Lebreton et al., 2015). Thus, when rating likeability, a strongly positive or negative reaction feels more certain to participants. Prompting confidence may highlight this association, encouraging participants to prefer the endpoints of the rating scale. Notably, we did not find evidence that the effect of eliciting confidence ratings was moderated by the average confidence associated with a judgment. This suggests that the difference in effects observed for subjective and objective tasks is unlikely to be fully accounted for by the sheer confidence associated with the type of judgments used in each task, though we cannot rule out entirely (and indeed suspect it is the case) that confidence may well be part of what produces the distinct effects between subjective and objective tasks.

#### Why would confidence ratings reduce extremity in objective tasks?

In contrast, for objective estimates, like dating a painting, metacognitive reflection might cause participants to recalibrate or second-guess themselves. Research on “confidence reactivity” in perceptual and cognitive tasks shows that asking for confidence can prompt people to collect slightly more evidence, raise their decision thresholds, and become more cautious (Li et al., 2024; Petrusic & Baranski, 2003). Indeed, participants in objective contexts may interpret the confidence question as a signal to be “more precise,” leading them to avoid the scale’s extremes if they lack strong knowledge (which, given the esoteric nature of the task, is likely to be the case for many of our participants). Consistent with this, we found that CR participants *did* converge on somewhat less extreme year estimates, though not necessarily more accurate ones, suggesting a nuanced effect on how they used the rating scale. In addition, the relative difficulty of dating paintings may have encouraged a more conservative response criterion, producing less extreme estimates. Thus, the reduction in polarization observed for objective judgments may partly reflect criterion setting rather than domain differences alone.

#### Metacognition and polarization

Our findings contribute to a complex picture of how metacognitive prompts can either reduce or heighten biased thought. The emerging pattern suggests that the effect of metacognitive prompts hinges critically on whether the task involves subjective or objective judgment. Subjective judgments, especially those lacking accountability or feedback, may see confidence ratings amplify initial intuitions and preferences, increasing extremity. Objective judgments, in contrast, may benefit from metacognitive prompts that induce more cautious responding. This task-based asymmetry could reconcile prior inconsistencies in the literature. Some interventions using metacognition have shown promise in lowering partisan bias, reducing negative stereotyping, or encouraging more careful belief updating (Reininger et al., 2020; Voelkel et al., 2023). In contrast, others have observed that making metacognitive ratings can impede deeper learning or reinforce preexisting beliefs (Double et al., 2025a; Mitchum et al., 2016). Indeed, dogmatic or highly extreme thinkers often exhibit reduced metacognitive sensitivity, failing to discount their confidence enough in the face of contradictory evidence (Rollwage et al., 2018). In that sense, confidence ratings risk strengthening the link between certainty and belief if participants are not motivated (or able) to reflect critically when these ratings are elicited.

In light of these results, interventions that aim to use metacognitive reflection to de-polarize attitudes should proceed with caution. Simply asking individuals, “How confident are you?” may backfire in some contexts, causing them to entrench more deeply in their immediate preferences or beliefs. Instead, interventions might incorporate more structured approaches that more specifically prompt deeper thought and provide encouragement for participants to challenge their own beliefs, for example, asking people to generate reasons why they might be wrong (Lord et al., 1984).

#### Limitations and future directions

The present study has several limitations which should be noted. First, the confidence ratings might have produced a more simplistic demand characteristic in participants. Specifically, participants might have inferred that they needed to give stronger ratings to justify their confidence. However, if this is the case we might have expected similar results in the objective ratings made in Experiment 3. Nonetheless, future studies may wish to examine different prompts (as well as control ratings) to examine this possibility. A second possibility is confidence-rating fatigue or irritation, particularly in subjective tasks, which could itself bias responses (e.g., toward endpoints to ‘justify’ the added effort). Future work could compare continuous vs. intermittent confidence prompts to test this possibility. In addition, because the present experiments used a between-subjects design, condition effects may reflect stable between-group differences rather than within-person reactivity. Future studies using interleaved or blocked within-subjects designs could provide stronger tests of causal mechanisms underlying confidence reactivity. Notably, reactivity effects have been shown in both between and within-subject designs (Double & Birney, 2024; Double et al., 2025b). It is worth noting, that within subject designs can be complicated to use in reactivity research as the effect of eliciting confidence ratings may spillover to other items (or blocks).

A methodological limitation of the present approach is that mean confidence was estimated at the stimulus level using data from the confidence-rating condition. This assumes that confidence in each stimulus is relatively stable across participants and conditions, which may not strictly hold. Because this approach collapses across individuals, it likely introduces random noise and reduces sensitivity to moderation effects. Nonetheless, this approach allows for an interpretable, stimulus-linked test of reactivity while preserving statistical independence between conditions.

Additionally, real-world polarization often unfolds over repeated exposures to like-minded or contrary evidence. Investigating whether confidence ratings exacerbate response polarization over multiple sessions remains a promising avenue for future research. It would also be informative to explore individual differences (e.g., dogmatism, political ideology, or need for closure) that could moderate the effects of confidence prompts.

#### Conclusion

These experiments highlight that trial-by-trial confidence ratings have domain-specific effects on the extremity of judgments. For subjective attitudes—such as art preferences or agreement with social statements—confidence ratings led to increased polarization; for objective judgments about painting completion years, confidence prompts reduced extremity. Together with prior studies on metacognitive reflection and biased belief updating, our results underscore that simply “measuring confidence” can be a notable intervention, but not always in the intended direction. Eliciting confidence in subjective domains risks reinforcing immediate affective responses, thereby raising the danger of rigid thinking and polarized positions. Effective de-polarization approaches likely require a more nuanced, guided form of self-reflection, one that encourages individuals to question their certainty rather than merely articulate it.

## Data Availability

Materials, data, and code are available on the Open Science Framework ([https://osf.io/ju73w/](https:/osf.io/ju73w/)).
